# Genetically-informed prediction of short-term Parkinson’s disease progression

**DOI:** 10.1038/s41531-022-00412-w

**Published:** 2022-10-28

**Authors:** Hossein J. Sadaei, Aldo Cordova-Palomera, Jonghun Lee, Jaya Padmanabhan, Shang-Fu Chen, Nathan E. Wineinger, Raquel Dias, Daria Prilutsky, Sandor Szalma, Ali Torkamani

**Affiliations:** 1 Scripps Research Translational Institute, La Jolla, CA 92037 USA; 2 Department of Integrative Structural and Computational Biology, Scripps Research, La Jolla, CA 92037 USA; 3 Takeda Development Center Americas, Inc., San Diego, CA 92121 USA; 4 Takeda Development Center Americas, Inc., Cambridge, MA 02139 USA

**Keywords:** Genomics, Clinical trial design, Predictive markers

## Abstract

Parkinson’s disease (PD) treatments modify disease symptoms but have not been shown to slow progression, characterized by gradual and varied motor and non-motor changes overtime. Variation in PD progression hampers clinical research, resulting in long and expensive clinical trials prone to failure. Development of models for short-term PD progression prediction could be useful for shortening the time required to detect disease-modifying drug effects in clinical studies. PD progressors were defined by an increase in MDS-UPDRS scores at 12-, 24-, and 36-months post-baseline. Using only baseline features, PD progression was separately predicted across all timepoints and MDS-UPDRS subparts in independent, optimized, XGBoost models. These predictions plus baseline features were combined into a meta-predictor for 12-month MDS UPDRS Total progression. Data from the Parkinson’s Progression Markers Initiative (PPMI) were used for training with independent testing on the Parkinson’s Disease Biomarkers Program (PDBP) cohort. 12-month PD total progression was predicted with an F-measure 0.77, ROC AUC of 0.77, and PR AUC of 0.76 when tested on a hold-out PPMI set. When tested on PDBP we achieve a F-measure 0.75, ROC AUC of 0.74, and PR AUC of 0.73. Exclusion of genetic predictors led to the greatest loss in predictive accuracy; ROC AUC of 0.66, PR AUC of 0.66–0.68 for both PPMI and PDBP testing. Short-term PD progression can be predicted with a combination of survey-based, neuroimaging, physician examination, and genetic predictors. Dissection of the interplay between genetic risk, motor symptoms, non-motor symptoms, and longer-term expected rates of progression enable generalizable predictions.

## Introduction

Parkinson’s disease (PD) is a slowly progressing neurodegenerative disorder classically characterized by the loss of dopaminergic neurons in the substantia nigra with resultant bradykinesia with resting tremor or rigidity^1,2^. Available treatments primarily act to restore dopamine levels (e.g., levodopa, COMT inhibitors, MAO-B inhibitors), stimulate dopaminergic neurons (e.g., dopamine agonists), or modify the symptoms of PD (e.g., anticholinergics, amantadine, deep brain stimulation). These treatments have not been shown to slow disease progression, which is characterized by varied motor and non-motor changes overtime^3^.

Recently, there has been interest in defining disease trajectories or subtypes in order to better diagnose, prognose, and treat PD^2,4–7^. While there are no formally recognized disease subtypes, data-driven methods are converging on disease states or subtypes characterized by differing severity and progression rate of motor symptoms and non-motor symptoms^2,4,5,7,8^. One approach to subtyping finds: (1) a slowly progressing, earlier onset, mild motor predominant disease subtype, (2) a rapidly progressing subtype with severe motor symptoms, cognitive impairment, and sleep disturbances, and (3) an intermediate subtype with no cognitive impairment but both moderate motor and non-motor symptoms^2,4,5^. A prodromal phase presumably precedes these subtypes with sleep disorder, urinary dysfunction, and other non-motor symptoms, though it has been recently suggested that the prodromal phase is also characterized by subtypes distinguished by motor, non-motor, and genetic subtypes^9^. Ultimately, whether these subtypes/states represent a single disease spectrum or distinct disease entities is unknown^10^, but it is clear that PD progression is variable with some recurrent patterns in motor and non-motor progression trajectories.

This heterogenous nature of PD hampers clinical research, with numerous agents failing to show neuroprotection through the slowing or modification of disease progression^11,12^. The possible underlying reasons for these failures are numerous and unclear, but thought to include issues with traditional clinical trial design, like too short a follow-up period in cohorts with disease that is too advanced for effective intervention^10^. These issues result in expensive and time-consuming clinical trials prone to failure with long timespans separating trial initiation and resultant learnings. Adaptable trials allowing for rapid iteration, learning, and failure may accelerate clinical research and development, but their implementation is challenged by slow disease progression without reliable and rapid readouts^10,13^. Here we attempt to identify short term PD progressors, with the goal of shortening the duration of clinical trials by selectively recruiting subjects who are destined to be short-term progressors. When progressors are enrolled, one can expect the experimental and control groups to diverge faster if the neuroprotective agent being tested is effective. The primary outcome in these clinical trials is progression of the MDS-UPDRS Total score.

In this light, we develop machine learning models, trained on the Parkinson’s Progression Markers Initiative (PPMI) cohort, and externally validated on the Parkinson’s Disease Biomarkers Program (PDBP) cohort. Our approach focuses on the prediction of short-term (12-month) PD progression using only baseline features collected at the time of study enrollment. Given the lack of specific markers for neuroprotection, clinical outcome measures are often used as trial outcomes, namely the Movement Disorder Society-sponsored Revision of the Unified Parkinson’s Disease Rating Scale (MDS-UPDRS). Thus, here we predict progression in MDS-UPDRS Total values, or the combined change of motor and non-motor impairment over time.

Most previous efforts to apply machine learning to PD and other neurodegenerative diseases have been focused on early detection and disease monitoring applications, especially for the differentiation of related disorders^14^. Several studies have attempted PD progression prediction, but very often focus on progression of a single motor (often MDS-UPDRS III) or non-motor (often cognitive decline) factor^15–20^, which can be useful for clinical management. For example, a previous study from The Parkinson’s Progression Markers Initiative found that lower baseline motor impairment as ascertained by physician-exam (MDS-UPDRS III), lower striatal DAT binding, and lower CSF amyloid-β_1–42_ were predictive of disease progression, especially motor progression^15^. Subtyping-based studies are more comprehensive in integrating and projecting motor and non-motor impairment but tend to focus on long-term clinical trajectories and outcomes. For example, Severson et al. define disease states capable of handling PD heterogeneity through comprehensive modeling, but not including genetic data. They derive disease states predictive of long-term (3–5 year) progression to severe disease^7^. Two other prior studies, discussed below, are most relevant to our work, focusing on either short-term progression or incorporating genetic predictors^18,21^.

Nguyen et al. use a combination of demographic, clinical, and neuroimaging features to predict short-term MDS-UPDRS Total disease severity in ElasticNet regression, Support Vector Machine, Random Forest, and Gradient Boosted decision tree frameworks, with a focus on dissecting the relation of neuroimaging markers with disease severity. Note that this study focuses on predicting future absolute disease severity, in contrast to our focus on change over time. In this scenario and in contrast to progression predictions described above, higher baseline impairment is predictive of future severe disease. They achieve good predictive 1-year accuracy (PPMI AUC 0.75) but with reduced generalizability in the external validation set (PDBP 0.69) except when identifying those individuals with the most severe disease^21^.

More relevant from a genetics perspective, Latourelle et al. focused on prediction of longer-term (3-years or longer) progression of motor impairment (combined MDS-UPDRS II and III) trajectories using a combination of demographic, clinical, genetic, and neuroimaging features^18^. They demonstrate the importance of genetic predictors, but also observe a loss in generalizability in the external validation dataset (*R*^2^ of 27% reduced to 9% overall), with most of the separation of slow and fast progressors observed at longer (4+ years) of follow-up. Their unbiased use of genome-wide individual genetic markers, while handled with appropriate care, is prone to overfitting due to population stratification.

Thus, our analysis is differentiated by the short time span of combined motor and non-motor (MDS-UPDRS Total) progression trajectories studied, the combination of predictive features employed including the use of multiple polygenic risk scores (Parkinson’s disease diagnosis^22^ and educational attainment^23^), and the multivariate, meta-predictive framework employed to achieve generalizable prediction of comprehensive PD progression. We demonstrate that our approach can (1) capture the heterogeneity of short-term progression trajectories that are likely hindering generalizability of more direct predictive strategies, (2) identify polygenic risk^24^ as important contributors to generalizable prediction, and (3) disentangle and characterize the conflicting baseline conditions (potentially subtypes) driving heterogenous disease progression. We suggest these models may be useful for planning adaptive trials without excluding disease subtypes in early trial phases.

## Methods

### Data

Data from the Parkinson’s Progression Markers Initiative (PPMI) and Parkinson’s Disease Biomarkers Program (PDBP) cohorts were accessed from the Accelerating Medicines Partnership: Parkinson’s Disease (AMP-PD) repository v1 release^25^. Data were accessed under AMP-PD Data Use Agreement and downloaded from https://amp-pd.org/. Data was collected prior to this study and accessed de-identified. Participants provided written informed consent for data sharing to the original PPMI and PDBP studies, under protocols approved by the Indiana University IRB (PPMI) and each PDBP center.

Individuals were included in this study if they had a diagnosis of “Parkinson’s Disease” or “Idiopathic Parkinson’s Disease” at baseline. In order to maximize the real-world utility in new prospective cohorts, all standardization and correction factors that are described below are defined in the PPMI training cohort and applied to both the PPMI training and PDBP testing cohorts.

### Definition of progressors vs non-progressors

MDS-UPDRS subpart values were first adjusted for medication treatment status by determining the average difference in MDS-UPDRS subpart values for treated vs untreated PPMI individuals across all timepoints and adding this average difference to the respective MDS-UPDRS subpart values of treated individuals. Due to the high level missingness for time-point specific treatment data, we considered an individual treated if they were treated at any point during the predicted follow-up period. The adjustment for medication to all MDS-UPDRS scores for treated individuals was: +0.67, +1.5, and +3.67 for MDS-UPDRS I, II, and III respectively. For this and all subsequent analyses, medication adjusted values are used. Given that we used a mixture of MDS-UPDRS responses on and off medication, we confirmed there was no association between levodopa, dopamine agonists, or other PD medication usage status and MDS-UPDRS subpart (Supplemental Table 1) or Total (Table 1) progression status in both PPMI and PDBP. Progressors vs non-progressors were defined based on the slope of their medication-adjusted MDS-UPDRS subpart values for each time period—from baseline to 12-, 24-, and 36-months post baseline and for each MDS-UPDRS subpart individually. Progressors are defined as those individuals with a slope greater than zero, non-progressors are defined as those individuals with an slope less than or equal to zero. MDS-UPDRS Total is defined as the sum of these subpart values, as normal, and progressors defined similarly by the slope of the MDS-UPDRS Total value. Ultimately, each individual is separately classified as a progressor or non-progressor across all time intervals (12- 24- and 36-months post baseline) and for each MDS-UPDRS subpart (I, II, and III), resulting in nine different progressor vs non-progressor classifications per individual.

**Table 1:** Baseline characteristics of 12-month MDS-UPDRS total progressors vs non-progressors.

Predictive feature	Progressors: Non-progressors (PPMI)	*P*-value	Progressors: Non-progressors (PDBP)	*P*-value
*Demographics*	64%: 36%	-	48%:52%	-
Sample size (*n*)	529	-	350	-
Age (years)	61.9 ± 0.5: 62.1 ± 0.61	0.033	59.4 ± 1.15: 60.0 ± 1.01	0.034
Sex (%F)	24%: 22%	-	60%: 73%	-
*Treatment status (%)*				
Levodopa	14% ± 0.02: 19% ± 0.03	0.312	75% ± 0.03: 76% ± 0.03	0.856
Dopamine agonist	10% ± 0.02: 13% ± 0.02	0.563	55% ± 0.04: 50% ± 0.04	0.434
Other PD medication	14% ± 0.02: 18% ± 0.03	0.479	74% ± 0.03: 74% ± 0.03	0.932
*Clinical instruments*				
MDS-UPDRS I	4.1 ± 0.26: 4.6 ± 0.36	0.204	4.1 ± 0.39: 4.6 ± 0.43	0.566
MDS-UPDRS II	3.5 ± 0.25: 4.2 ± 0.34	0.043	4.7 ± 0.5: 5.6 ± 0.56	0.355
**MDS-UPDRS III**	**15.9** ± **0.53: 21.0** ± **0.73**	**<0.001**	**19.1** ± **0.94: 24.4** ± **1.05**	**<0.001**
**MDS-UPDRS Total**	**23.6** ± **0.8: 29.9** ± **1.07**	**<0.001**	**28.0** ± **1.51: 34.8** ± **1.71**	**0.002**
Hoehn & Yahr	1.5 ± 0.03: 1.7 ± 0.04	<0.001	2.03 ± 0.04: 2.1 ± 0.05	0.415
SE-ADL	94.0 ± 0.42: 93.7 ± 0.52	0.478	88.5 ± 0.93: 87.4 ± 1.02	0.568
MoCA	27.1 ± 0.15: 27.2 ± 0.2	0.013	26.0 ± 0.24: 26.1 ± 0.25	0.628
ESS				
*DaTScan neuroimaging*				
sbr_caudate_r	2.1 ± 0.04: 2.0 ± 0.06	0.06	-	-
sbr_caudate_l	2.1 ± 0.04: 2.0 ± 0.06	0.048	-	-
sbr_putamen_r	0.9 ± 0.03: 0.9 ± 0.05	0.02	-	-
sbr_putamen_l	0.9 ± 0.03: 0.9 ± 0.05	0.018	-	-
*Genetics*				
Parkinson’s disease PRS (ave)	0.004 ± 0.0: 0.005 ± 0.0	0.229	0.0026 ± 0.0: 0.0025 ± 0.0	0.534
Educational attainment PRS (ave)	0.0005 ± 0.0: 0.0004 ± 0.0	0.594	0.0005 ± 0.0: 0.0004 ± 0.0	0.091
Monogenic risk (%)	32%: 40%	-	9%: 9%	-

Features consistently different between progressors and non-progressors in both PPMI and PDBP are bolded. See Supplemental Tables 2–3 for a more detailed comparison. All comparisons are made with the non-parametric Wilcoxon rank sums test.

Note that the above definition is equivalent to simply defining progressors as those individuals with an increase in their unadjusted MDS-UPDRS values over the given time interval. Medication adjustment does not change these classifications as it is applied uniformly across all timepoints. However, we used these slope values, as well as MDS-UPDRS subpart slopes calculated over internal time intervals (e.g., 12 to 24 months), in a number of exploratory agglomerative clustering analyses, which also included absolute baseline MDS-UPDRS values, in order to determine whether data driven approaches interrogating the shape of the progression trajectory may be used to better define progressors vs non-progressors (data not shown). Ultimately, it was determined that clustering-based approaches largely agreed with the simpler definition, with more bias observed in the baseline ages of progressors vs non-progressors when defined by clustering vs simple MDS-UPDRS score changes. Therefore, we proceeded with this straightforward definition of PD progression.

### Definition of predictors

We considered all available demographic, genetic, survey instrument, clinical status, clinical examination, and neuroimaging data as potential predictive features. Initially, predictive features were eliminated if they had >50% missingness. Otherwise, missing values were imputed simply using item averages for continuous predictors, median values for ordinal predictors, and the most common value for categorical predictors. After feature selection, the remaining selected features had <5% missingness prior to imputation with an overall average level of missingness being <1%. Features were ranked for predictive importance using Shapley Additive Explanations (SHAP) values^26^ using the initial modeling approach described in *Predictive Model Development* below. The top 25 predictive features were used in final modeling. Predictive features included: (1) baseline age, gender, and education status, (2) baseline MDS-UPDRS I, II, and III subpart and Total values as well as the responses to individual subpart elements, (3) Montreal Cognitive Assessment (MoCA) subscores and total score as well as the responses to individual score elements, (4) Modified Schwab and England Percent Activities of Daily Living (SE-ADL), 5) Dopamine Transporter Scan (DaTScan) striatum binding ratios (left and right caudate and putamen ratios), (6) Hoehn and Yahr Scale, and 7) genetic predictors which included; a 90-SNP polygenic risk score for Parkinson’s disease diagnosis^22^, a 763-SNP polygenic score for educational attainment^23^, and monogenic mutation status for *GBA*, *LRRK2*, and *SNCA* as cataloged in AMP-PD and collapsed into a single binary variable per gene and as a single combined variable. Predictive features are mean-standardized using factors derived only from the training dataset and applied to the testing dataset. We use a standard scaler for this purpose, subtracting the mean and scaling to unit variance.

The Epworth Sleepiness scale was found to contribute minimal additional predictive power and in conjunction with MDS-UPDRS subpart components and excluded from use. DaTScan imagining values were only present in PPMI and thus only present in train-test split models of PPMI. Also note that whether responses were provided by the patient or caregiver are encoded in the “info source” feature in AMP-PD, but ultimately not selected as an important feature. Summary comparisons of these predictive features and their comparison in progressors vs non-progressors are presented in Table 1 with the full set of MDS-UPDRS and MoCA elements presented in Supplemental Table 2 (PPMI) and Supplemental Table 3 (PDBP). All feature comparisons are made using the nonparametric Wilcoxon Rank Sum Test. All features are labeled corresponding to the AMP-PD research data dictionary “data element” column.

### Polygenic score calculation

Polygenic scores were calculated using whole genome sequencing data processed and quality-controlled by the AMP-PD consortium^25^. Individuals of European ancestry were selected as those individuals with genetic principal component values within seven standard deviations of the average for the first six principal components of European 1000 Genomes Phase 3 reference panel individuals^27^. Principal components were calculated using variants with a call rate>95%, Hardy–Weinberg equilibrium *p*-value > 1e−15, and minor allele frequency >1%. These variants were pruned using a window size 50, step 5, and *r*^2^ 0.2 in PLINK^28^. For the PD PRS, beta estimates for 90 SNPs independently associated with PD in the largest recent GWAS meta-analysis^22^ were used to calculate individual level PRSs using the standard weighted allele-counting approach^29^. Similarly, for educational attainment, beta estimates for 763 SNPs independently associated with educational attainment^23^ were used to calculate individual level PRSs using the standard weighted allele-counting approach^29^.

### Predictive model development

PPMI data was split into 75% training and 25% testing data for the development of initial unoptimized models for the prediction of MDS-UPDRS I, II, and III progression status at 12-months using XGBoost^30^, a simple feed forward neural network (FFNN), balanced random forest^31^ (BRF), and logistic regression (LR). A further split of the PPMI training dataset into a 75% training and 25% validation data subset was performed for feature selection by SHAP values^26^. To calculate SHAP values an explainer model is tuned on this internal training data subset to drive feature selection and importance. After feature selection, fully trained models are applied to the full PPMI dataset to generate final importance plots using the SHAP function of the sci-kit learn library. Global summary plots are then generated using the summary plot function in the SHAP library.

For initial models, XGBoost models were clearly superior, achieving F1 scores of 0.70 for MDS-UPDRS I vs. 0.68, 0.55, and 0.54 for FFNN, BRF, and LR respectively, 0.78 for MDS-UPDRS II vs 0.63, 0.69, and 0.68 for FFNN, BRF, and LR respectively, and 0.77 for MDS-UPDRS III vs 0.70, 0.71, 0.69 for FFNN, BRF, and LR respectively. Similar relative results were observed for 24- and 36-month prediction. We also attempted quantitative predictions of progression rate but found that these predictions were difficult and led to poorer performance overall. Therefore, we limited additional model development to the binary classification problem in the XGBoost predictive framework.

Refined XGBoost models were then prepared for 12-, 24-, and 36-month prediction with Optuna hyperparameter optimization^32^ using the default random sampler. Tuning parameters and ranges include: lambda_par = [1e,1–8], alpha = [1e,1–8], subsample = [0.5, 1], colsample_bytree = [0.5, 1], scale_pos_weight = [0.8, 1.2], max_depth = [6,36], min_child_weight = [1,10], eta = [1e,1–8] gamma = [1e,1–8], grow_policy = [“grow_policy”, [“depthwise”, “lossguide”]], sample_type = [“sample_type”, [“uniform”, “weighted”]], normalize_type = [“normalize_type”, [“tree”, “forest”]], rate_drop = [1e,1–8]

skip_drop = [1e,1–8].

Only baseline features are used in these predictions, regardless of the timespan to the outcome. Final models were built by splitting PPMI into a 75% training and 25% validation set. The training set is further split 75%:25% into a training and testing set for feature selection. Thus, feature selection is evaluated on the hold-out test set and validated on the validation set.After feature selection, final training is performed on the full PPMI dataset and tested on the independent PDBP dataset. Stratified random sampling was used when splitting the data to maintain the balance of progressors vs non-progressors (see Table 1). Independent testing is of greatest interest, though due to the lack of DaTScan data in PDBP, DaTScan utility can only be evaluated in the PPMI train-test split models. Performance of these initial models are reported in Table 3.

### Meta-prediction

The output predictions of initial models predicting individual MDS-UPDRS sub-parts at 12-, 24-, and 36- months are subsequently used as features for final meta-prediction. Individual subpart model predictions across the different follow-up time periods are input into an XGBoost meta-predictor, in conjunction with the initial baseline features, for the final meta-prediction of 12-month MDS-UPDRS Total progression status.

### Model performance

Model performance is reported using F1 scores for initial models with the addition of ROC AUC and PR AUC for final meta-prediction models. See Table 3 and Table 4 for confidence intervals on reported performance metrics.

### Reporting summary

Further information on research design is available in the Nature Research Reporting Summary linked to this article.

## Results

### Characteristics of progressors vs non-progressors

The target prediction is short term PD progression status, defined as those individuals with a positive slope in their MDS-UPDRS Total value from baseline to 12-months post baseline. A comparison of the demographic, clinical, neuroimaging, and genetic characteristics of 12-month progressors vs non-progressors in both PPMI and PDBP is provided in Table 1. Considering consistent trends observed in both PPMI and PDBP, while some measures demonstrated marginal differences between progressors and non-progressors, only the baseline MDS-UPDRS III and Total scores were significantly different at baseline, with progressors displaying less baseline impairment overall. This trend was also apparent, but not significant, for MDS-UPDRS I, MDS-UPDRS II, and the Hoehn & Yahr functional impairment values. This observation also extends to individual subpart components, with components of MDS-UPDRS III showing the greatest differences in progressors vs non-progressors, again with progressors consistently displaying less baseline impairment (Supplemental Tables 2, 3).

Given the relatively weak relation of 12-month MDS-UPDRS I and II progression status with 12-month MDS-UPDRS Total progression status, we evaluated the overlap in subpart and total progression status directly (Table 2). In general, MDS-UPDRS subpart progression statuses overlapped moderately with one another (~60% overlap between subparts). Progression in any individual subpart overlapped more strongly with 12-month MDS-UPDRS Total progression, with the larger MDS-UPDRS III component showing greatest overlap (87%) with 12-month MDS-UPDRS Total progression. Thus, progression across all MDS-UPDRS subparts contribute with varying degrees to short term MDS-UPDRS Total progression, partially explained by their unequal contributions to the total score but supporting the notion that PD progression is characterized by heterogeneous progression of motor and non-motor changes overtime.

**Table 2:** Heterogeneity in 12-month MDS-UPDRS subpart progression.

	MDS-UPDRS I	MDS-UPDRS II	MDS-UPDRS III	MDS-UPDRS Total
MDS-UPDRS I	100%	58%	53%	62%
MDS-UPDRS II	-	100%	61%	71%
MDS-UPDRS III	-	-	100%	87%
MDS-UPDRS Total	-	-	-	100%

Overlap across MDS-UPDRS subparts and Total progression status, as measured by Jaccard similarity. This matrix is symmetric thus lower diagonal values are excluded.

**Fig. 1:**
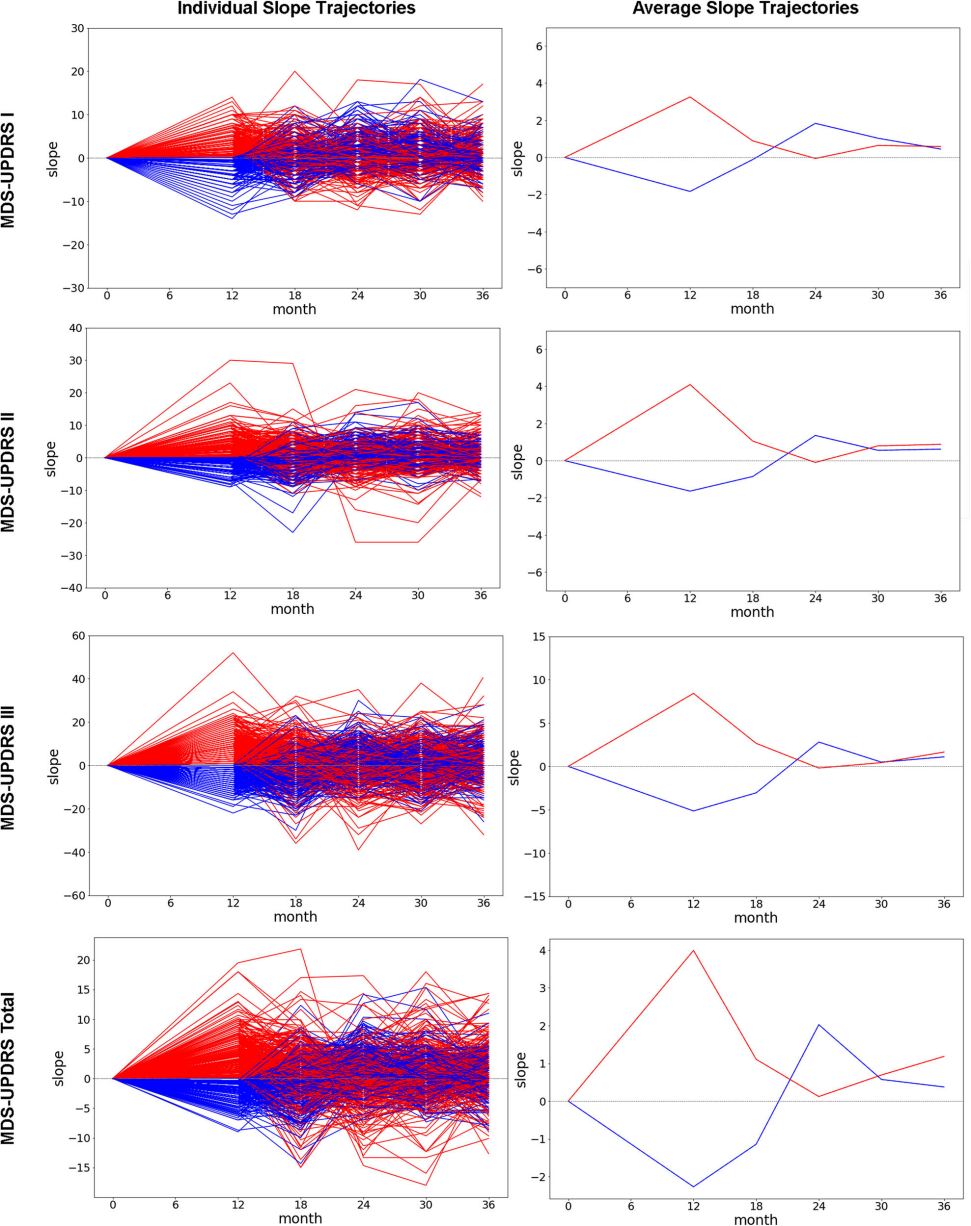
MDS-UPDRS annualized trajectories. Annualized MDS-UPDRS subpart and total slopes for the PPMI cohort. Each value corresponds to the slope of the line connecting the each MDS-UPDRS component value with its respective value preceding it by 12 months. For example, the 18-month timepoint is the slope of the difference between 6-month and 18-month MDS-UPDRS values. Individual trajectories are plotted on the left, with average values provided on the right. Lines are color coded based upon their baseline to 12-month MDS-UPDRS Total progression status (red: progressors, blue: non-progressors).

This heterogeneity in short-term subpart progression status additionally manifests as heterogeneity in longer term individual-level progression trajectories. After 12-months post-baseline, initial progressors tend to slow or even reverse their trajectory from 12 to 24 months, while initial non-progressors tend to convert to slow progressors in the 12 to 24-month timeframe (Fig. 1, right panels). From 24-months onward both groups progress gradually across all subparts on average, with a slight bias in increased motor progression (MDS-UPDRS II and III) for initial 12-month progressors. MDS-UPDRS Total progression is similar, but with initial 12-month progressors characterized by increased overall progression rates in the long-run. Significant variability is apparent in individual level progression trajectories (Fig. 1, left panels).

### Direct prediction of PD progression

Given the heterogeneity observed in individual level trajectories, and the inconsistency of MDS-UPDRS subpart progression contributions to total progression, we reasoned that direct 12-month MDS-UPDRS Total progression may be difficult to generalize. As suspected and observed in prior studies^18,21^, developing models directly predicting 12-month MDS-UPDRS Total progression performed with reduced generalizability when trained on PPMI and tested on PDBP (12-month MDS-UPDRS Total F-measure 0.74 on PPMI falling to 0.71 for PDBP). In contrast, generalizability was observed for 12-month MDS-UPDRS subpart progression predictions, and further improved accuracy and generalizability was observed for longer term 24- and 36-month progression prediction across MDS-UPDRS subparts and MDS-UPDRS Total (Table 3, top). We theorized that longer term progression predictions would be more robust relative to the direct prediction of 12-month subpart progression. Before describing how this finding is used to improve the generalizability and accuracy of 12-month MDS-UPDRS Total progression prediction, we characterize the features driving individual MDS-UPDRS subpart progression predictions.

**Table 3:** Accuracy of direct and meta-prediction of MDS-UPDRS I, II, III, and total progression.

	PPMI train-test split	PDBP independent test
Direct prediction		
12-month MDS-UPDRS I	0.70 ± 0.04	0.70 ± 0.05
12-month MDS-UPDRS II	0.69 ± 0.04	0.70 ± 0.05
12-month MDS-UPDRS III	0.74 ± 0.04	0.73 ± 0.05
12-month MDS-UPDRS Total	0.74 ± 0.04	**0.71** ± 0.05
24-month MDS-UPDRS I	0.70 ± 0.04	0.69 ± 0.05
24-month MDS-UPDRS II	0.73 ± 0.04	0.72 ± 0.05
24-month MDS-UPDRS III	0.76 ± 0.04	0.74 ± 0.05
24-month MDS-UPDRS Total	0.75 ± 0.04	0.73 ± 0.05
36-month MDS-UPDRS I	0.76 ± 0.04	0.75 ± 0.05
36-month MDS-UPDRS II	0.72 ± 0.04	0.74 ± 0.05
36-month MDS-UPDRS III	0.74 ± 0.04	0.77 ± 0.04
36-month MDS-UPDRS Total	0.67 ± 0.04	0.74 ± 0.05
Meta-prediction		
12-month MDS-UPDRS Total	0.77 ± 0.04	**0.75** ± 0.05
24-month MDS-UPDRS Total	0.76 ± 0.04	0.75 ± 0.05
36-month MDS-UPDRS Total	0.77 ± 0.04	0.73 ± 0.05

**Fig. 2:**
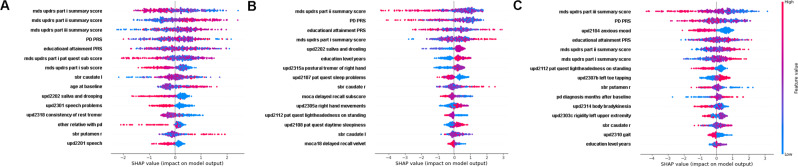
12-month MDS-UPDRS subpart and Total predictive feature importance. Shapley feature importance plots for 12-month MDS-UPDRS I (**A**), II (**B**), and III (**C**) subpart progression prediction. Features are ranked for most to least important (top to bottom). Coloration depicts the value for each feature (red = high values, purple = average values, blue = low values). The impact on prediction at the individual-level is indicated by the points, where points to the right indicate increased importance for prediction of progressor status, and points to the left indicate increased importance for the prediction of non-progressor status. See Fig. 3B for the Shapley plot for MDS-UPDRS Total progression prediction.

**Fig. 3:**
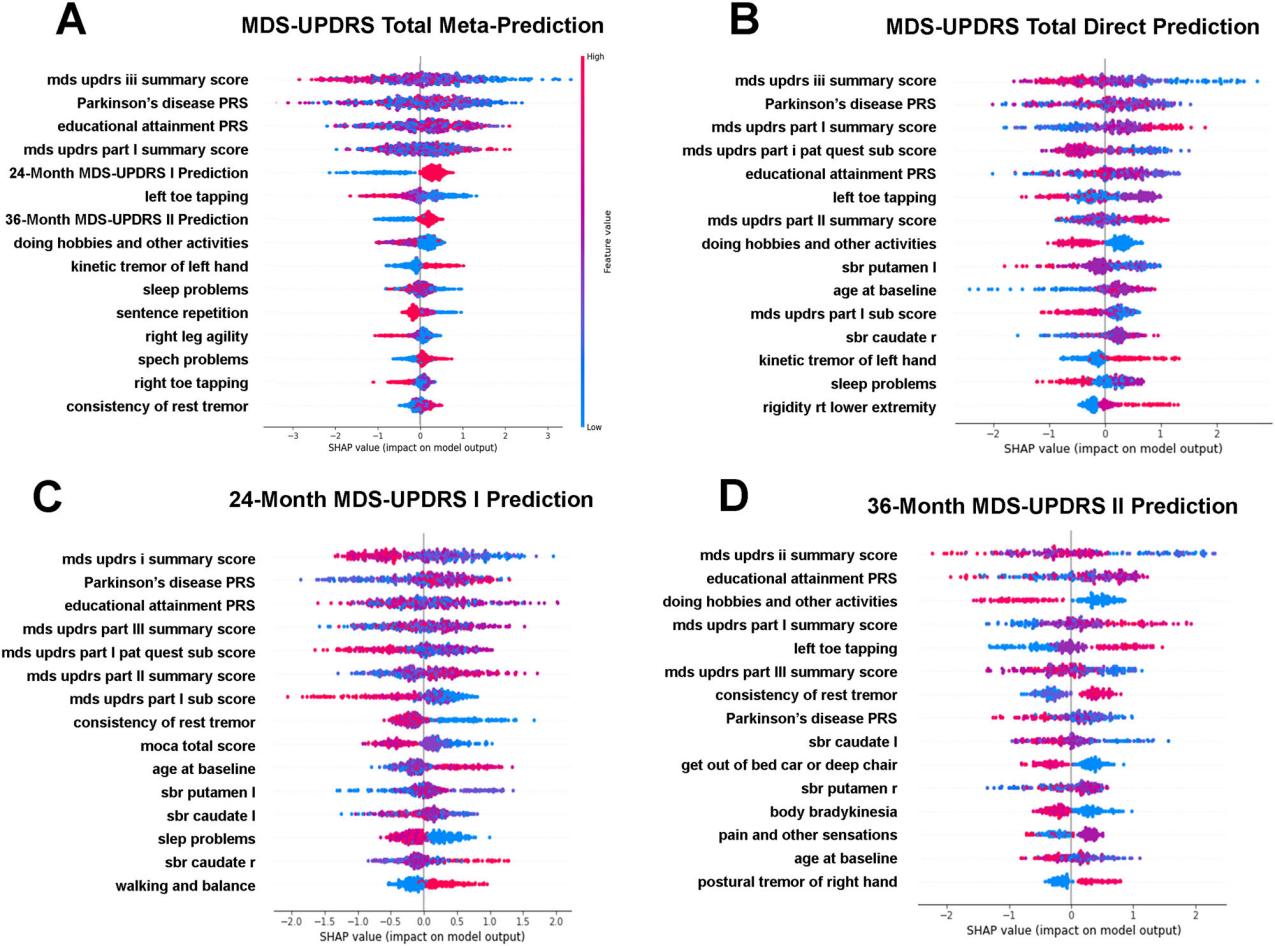
12-month MDS-UPDRS total meta-predictor feature importance. Shapley feature importance plots for direct (**B**) and meta-prediction (**A**) of 12-month MDS-UPDRS Total progression prediction. Shapley plots for important meta-features for meta-prediction of 12-month MDS-UPDRS Total progression status are shown in **C** 24-month MDS-UPDRS I prediction, and **D** 36-month MDS-UPDRS II prediction. Features are ranked for most to least important (top to bottom). Coloration depicts the value for each feature (red = high values, purple = average values, blue = low values). The impact on prediction at the individual-level is indicated by the points, where points to the right indicate increased importance for the prediction of progressor status, and points to the left indicate increased importance for the prediction of non-progressor status.

**Fig. 4:**
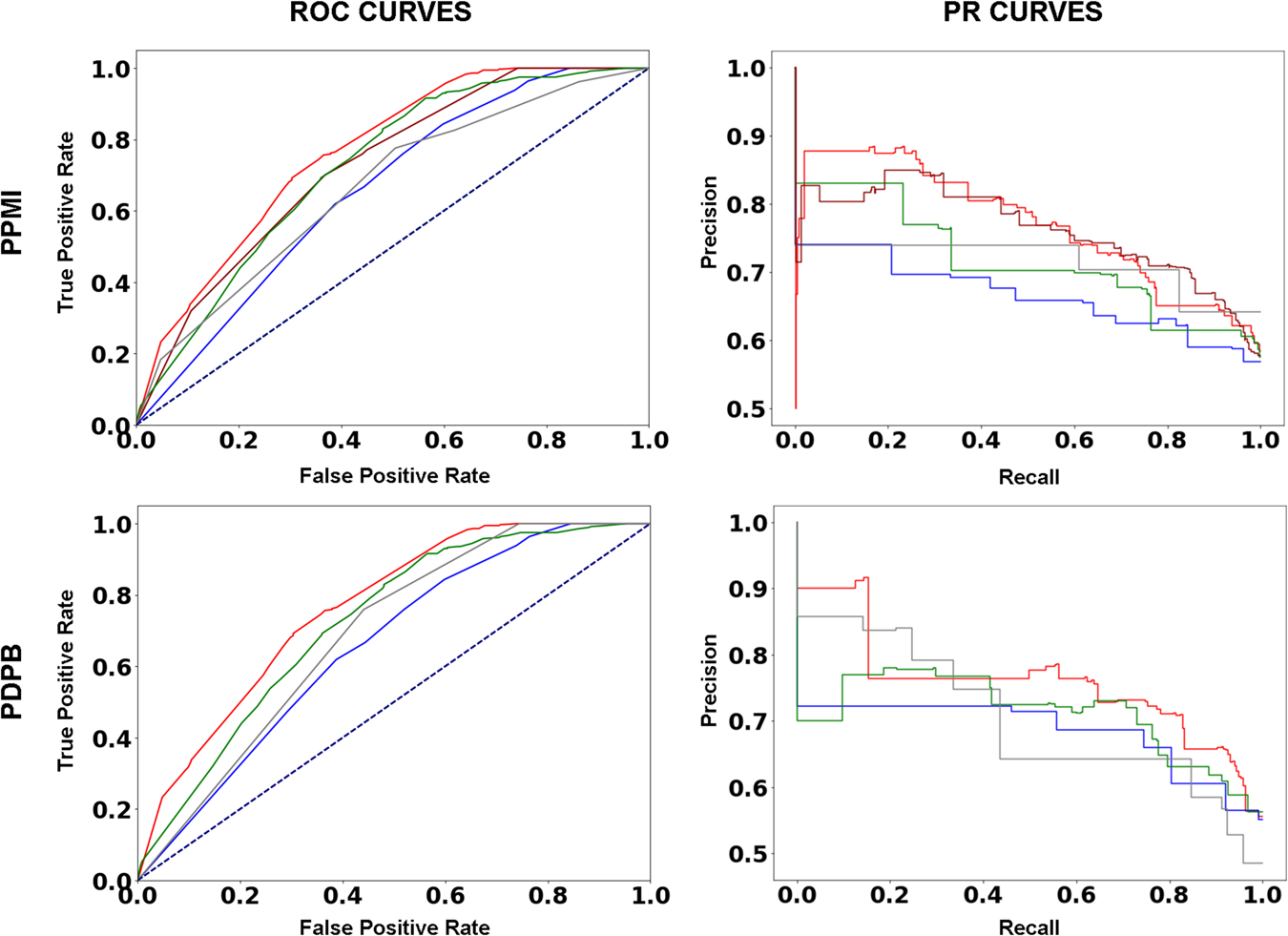
12-month MDS-UPDRS Total meta-predictive performance and feature class contributions. Performance of 12-month MD-UPDRS Total progression meta-prediction as measured by area under (AUC) receiver operating characteristic (ROC; left) and Precision-Recall (PR; right) curves. PPMI train-test split (top) and independent testing on PDBP (bottom) are presented. Curves are presented for full models (red), without genetic features (blue), without physician examination (MDS-UPDRS III) (gray), without survey-based features (MoCA, MDS-UPDRS I and II) (green), and without imaging features (magenta, PPMI only). See Table 4 for ROC AUC and PR AUC values.

F-measure accuracy of direct (single model) MDS-UPDRS I, II, III, and Total progression prediction (top) and F-measure accuracy of MDS-UPDRS Total meta-prediction (bottom). PPMI is used for training in all cases. Train-test split is 75% training and 25% testing with stratified sampling. See Fig. 2 for feature importance for MDS-UPDRS I, II, and III direct prediction and Fig. 3 for feature importance for MDS-UPDRS Total meta-prediction. ROC and PR curves for meta-prediction are provided in Fig. 4.

Shapley plots depicting the importance of features for 12-month MDS-UPDRS subpart progression predictions are presented in Fig. 2. Baseline MDS-UPDRS subpart measures were universally important predictors of progression across all subparts. A lower degree of baseline impairment for each MDS-UPDRS subpart was predictive of its respective 12-month progression. This relationship is reversed for baseline MDS-UPDRS subparts that are not the target of 12-month progression prediction. For example, a low baseline MDS-UPDRS I impairment, in conjunction with a greater degree of MDS-UPDRS II and III baseline impairment is predictive of future MDS-UPDRS I progression (Fig. 2A). This is true for MDS-UPDRS II and III 12-month progression status as well—lower baseline impairment of motor measures are predictive of their respective 12-month progression status, while higher baseline impairment of the non-target MDS-UPDRS I non-motor measure is predictive of 12-month motor progression status (Fig. 2B, C). In other words, progression for any individual MDS-UPDRS subpart is, on average, characterized by a “catch-up” effect in individuals with reduced baseline impairment of the target MDS-UPDRS subpart and higher baseline impairment in the non-target MDS-UPDRS subparts. These findings also demonstrate that the optimal conditions for short term progression of motor vs non-motor symptoms conflict with one another—i.e., the baseline conditions for simultaneous “catch-up” effects across motor and non-motor symptoms are in conflict.

Genetic risk is also universally important—being the second most important feature for self-reported MDS-UPDRS I and II progression status, and most important feature for prediction of the physician exam-based MDS-UPDRS III 12-month progression status. While the link between PD polygenic risk and non-motor progression appears complex (Fig. 2A), higher PD polygenic risk is universally associated with 12-month motor (MDS-UPDRS II and III) non-progression. This is also true for monogenic risk status (Table 1) where monogenic risk is associated with non-progression (or slower progression in the long-term), as previously noted for *LRRK2*^33^.

The polygenic educational attainment score follows as the next most important predictor, however, the relationship with progression status is complex. A clearer signal becomes apparent in meta-prediction of MDS-UPDRS Total progression, described later (Fig. 3). Cognitive impairment (MoCA Delayed Recall and Attention) is associated with lack of progression in self-reported measures (MDS-UPDRS I and II) but is not relevant for physician exam-based (MDS-UPDRS III) progression.

### Meta-prediction of PD progression

Given the previously described generalizability and accuracy of individual MDS-UPDRS subpart progression predictions, especially over longer follow-up durations, we evaluated whether these superior predictions could be combined to improve the generalizability and accuracy of short term MDS-UPDRS Total progression prediction. Indeed, meta-prediction of MDS-UPDRS Total progression was superior to direct MDS-UPDRS Total progression prediction (Table 3). 12-month MDS-UPDRS Total progression prediction on the independent PDBP testing set improved from a F-measure 0.71 for direct prediction (Table 3, top) to a F-measure of 0.75 with meta-prediction (Table 3, bottom). This meta-prediction model achieves a ROC AUC 0.74 and PR AUC 0.73 in the independent PDBP testing set (Fig. 4). Further improvements in longer term MDS-UPDRS Total progression prediction were also achieved (Table 3, top vs bottom).

Shapley plots depicting the feature importance for 12-month MDS-UPDRS Total progression meta-prediction and direct prediction are presented in Fig. 3A, B. Overall, the important predictive features remain similar, with the most important feature for both direct and meta-prediction being low baseline MDS-UPDRS III impairment. Similarly, MDS-UPDRS II impairment remains important in both direct and meta-prediction, but with the importance of baseline MDS-UPDRS II impairment replaced with the prediction of its progression at 36-months in meta-prediction. MDS-UPDRS I impairment also remains important in both direct and meta-prediction, but with the prediction of its progression at 24-months being added in addition to its baseline state in meta-prediction (Fig. 3A, B).

The two new meta-features, prediction of 24-month MDS-UPDRS I progression and 36-month MDS-UPDRS II progression can be observed to capture some opposing signals (Fig. 3C, D). For example, baseline impairment of all MDS-UPDRS subparts have opposing directions in these two meta-features. Similarly, age at baseline and the PD polygenic risk score have opposing effects for the positive prediction of progression. The combination of these two meta-features effectively capture the primary conflicting baseline signals for short-term motor vs non-motor progression.

**Fig. 5:**
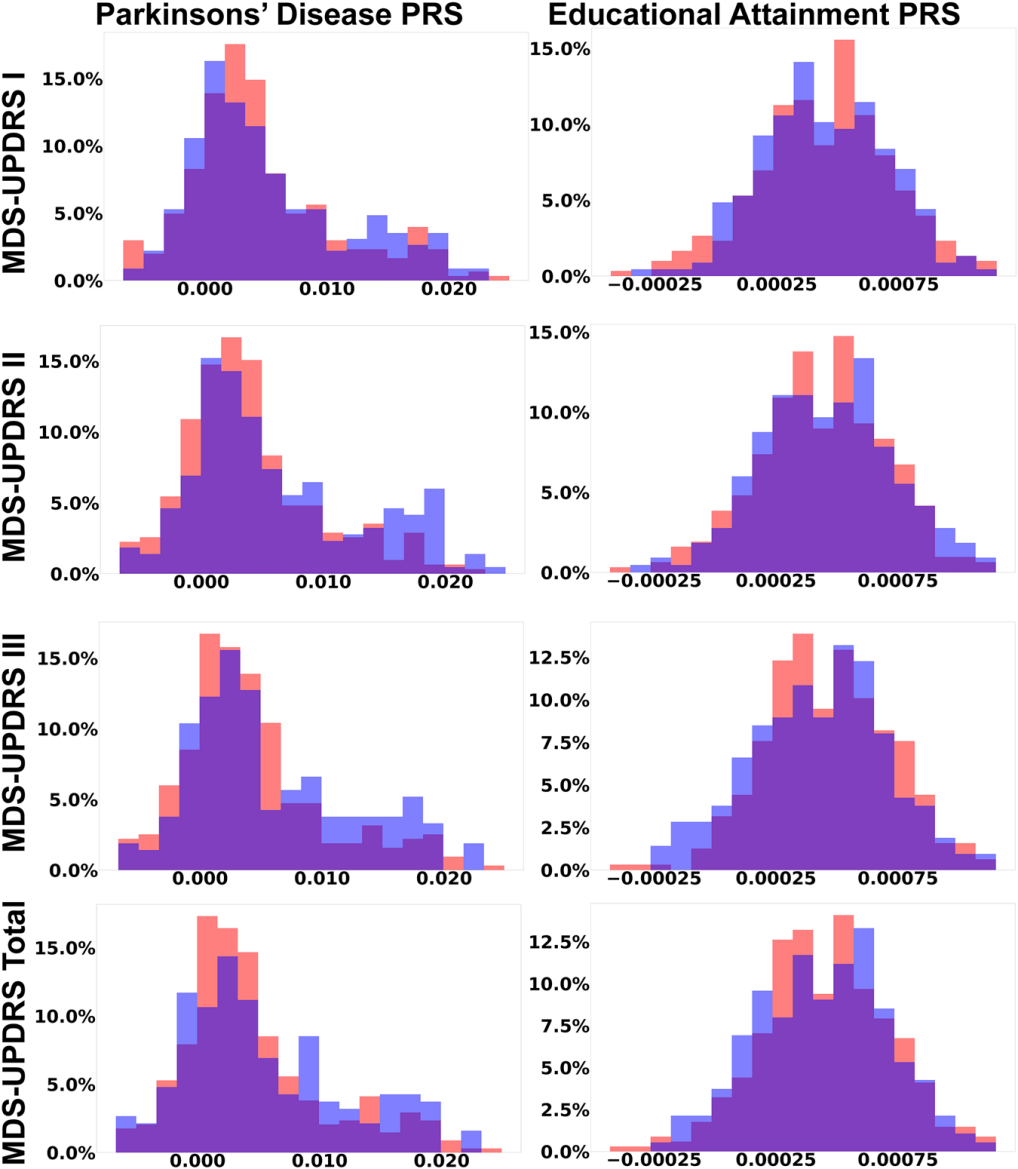
Distribution of polygenic scores in relation to MDS-UPDRS subpart and total progression. Distribution of polygenic scores for Parkinson’s disease diagnosis (left column) and Educational attainment (right column), by MDS-UPDRS subparts and Total progressors (red) and non-progressors (blue). Distribution overlaps are colored purple. The Parkinson’s disease PRS has a right tail that is more prominent in non-progressors, likely capturing a genetic subgroup of Parkinson’s disease due to high polygenic risk. The Educational attainment PRS is normally distributed with a minor left shift in the distribution for motor non-progressors.

As a primary predictive feature, higher PD polygenic risk is more clearly associated with non-progression in meta vs direct 12-month MDS-UPDRS Total prediction (Fig. 3A, B), being the 2nd most important predictor in both cases. This is likely primarily due to the association between higher PD polygenic risk and slower motor progression, as can be observed in the right-shift of the PD polygenic risk score distribution for MDS-UPDRS II, MDS-UPDRS III, and MDS-UPDRS Total non-progressors relative to progressors (Fig. 5, left). This shift is statistically significant for MDS-UPDRS II (*p*-value <0.001) and MDS-UPDRS III (*p*-value = 0.009), but not MDS-UPDRS I (*p*-value = 0.41) or MDS-UPDRS Total (*p*-value = 0.23). Monogenic risk does not rank among the most important features for short-term PD progression prediction.

Interestingly, a higher educational attainment polygenic score is more clearly associated with progression status in the meta-prediction framework relative to direct prediction (Fig. 3A, B). This association is most apparent in the left-shift of the educational attainment polygenic score for MDS-UPDRS III and MDS-UPDRS Total non-progressors relative to progressors (Fig. 5, right). At the population level, the effect of the PD and educational attainment polygenic score distribution shifts are subtle and not significant, but overall the educational attainment polygenic score is the 4th most important predictive features overall (Fig. 3A) as well as being in the top 3 most important features in the meta-features (Fig. 3C, D).

### Sensitivity of predictions to feature removal

To further validate the relative importance of predictive features, we removed genetic, neuroimaging, survey-based, and physician exam-based predictive features and evaluated the impact on 12-moth MDS-UPDRS total meta-prediction accuracy (Fig. 4). Removing genetic predictors led to the greatest loss in accuracy both when tested on PDBP; ROC AUC 0.74 vs 0.66, PR AUC 0.73 vs 0.66, and when performing train-test splitting on PPMI; ROC AUC 0.77 vs 0.66, PR AUC 0.76 vs 0.68 (Table 4). Removing physician exam-based predictive features (MDS-UPDRS III, Hoehn and Yahr staging, and contributing components) led to the next greatest loss in accuracy when tested on PDBP; ROC AUC 0.74 vs 0.69, PR AUC 0.73 vs 0.71, and when performing train-test splitting on PPMI; ROC AUC 0.77 vs 0.67, PR AUC 0.76 vs 0.71. Removing survey-based predictive features (MoCA, MDS-UPDRS I and II and contributing components) led to the third-most greatest loss in accuracy when tested on PDBP; ROC AUC 0.74 vs 0.72, PR AUC 0.73 vs 0.72, and when performing train-test splitting on PPMI; ROC AUC 0.77 vs 0.72, PR AUC 0.76 vs 0.72. And removing neuroimaging-based predictive features (DaTScan) led to the least reduction in accuracy, only testable when train-test splitting PPMI; ROC AUC 0.77 vs 0.73, PR AUC 0.76 vs 0.73 (Table 4). Full confusion matrices from these results are presented in Supplemental Table 4. Additional accuracy metrics (sensitivity, specificity, positive predictive value, negative predictive, and F1-score) are provided in Supplemental Table 5.

**Table 4:** Feature component accuracy of 12-month MDS-UPDRS total meta-prediction.

	PPMI train-test split		PDBP independent test	
	ROC AUC	PR AUC	ROC AUC	PR AUC
Full meta-prediction	077 ± 0.04	0.76 ± 0.04	0.74 ± 0.05	0.73 ± 0.05
No genetics	0.66 ± 0.04	0.68 ± 0.04	0.66 ± 0.05	0.66 ± 0.05
No physician exam	0.67 ± 0.04	0.71 ± 0.04	0.69 ± 0.05	0.71 ± 0.05
No surveys	0.72 ± 0.04	0.72 ± 0.04	0.72 ± 0.05	0.72 ± 0.05
No imaging	0.73 ± 0.04	0.73 ± 0.04	-	-

ROC AUC and PR AUC 12-month values for 12-month MDS-UPDRS Total progression prediction overall (full meta-prediction) and with feature class removal. PPMI is used for training in all cases. Train-test split is 75% training and 25% testing with stratified sampling. Note that removal of genetic features results in the greatest decline in predictive accuracy. ROC and PR curves for meta-prediction are provided in Fig. 4.

## Discussion

We demonstrate that short-term, generalizable, and comprehensive predictions of PD progression are possible by using a meta-predictive XGBoost framework. It is interesting that an out-of-the-box implementation of XGBoost is not able to completely overcome the heterogeneity of motor vs non-motor PD progression despite being an ensemble learning approach comprised of many decision trees^30^. This is likely due to the dominance of motor progression in MDS-UPDRS Total progression, both through the outsized contribution of MDS-UPDRS III to the Total score and the high-level similarity of MDS-UPDRS II and III progression features. Other tested statistical and machine learning models demonstrate inferior performance on even the simpler MDS-UPDRS subpart prediction tasks.

Like prior studies^15,18,21,34^, we find that low baseline MDS-UPDRS subpart impairment is among the most important predictors of progression status, but unlike prior studies we demonstrate how meta-prediction leads to more accurate and generalizable predictions through both the projection of long-term motor and non-motor impairment trajectories and the disentanglement of the conflicting baseline conditions leading to motor vs non-motor progression. While all PD individuals eventually progress at a similar rate at longer follow-up time periods, the rate and order of short-term symptom progression differs across individuals depending upon their baseline state, leading to differing and more rapid “catch-up” effects in motor vs non-motor symptoms. Thus, the superiority of meta-prediction is potentially due to the capture of motor vs non-motor PD progression subtypes, as well as aspects of the progression of the prodromal phase given the high importance of sleep disturbances in the meta-prediction framework. In addition, it appears that short term PD progression predictions benefit from the inclusion of more robust predictions of longer-term progression. This point is supported by the fact that 24-month MDS-UPDRS subpart I and 36-month MDS-UPDRS subpart II progression predictions are ultimately selected in the meta-prediction framework over the 12-month progression prediction alternatives available during meta-prediction feature selection.

We also uniquely demonstrate the importance of polygenic scores^24^ for Parkinson’s disease diagnosis^22^ and educational attainment^23^ for PD progression prediction. Genetic studies have previously identified individual markers, especially for *GBA* and *APOE*, in PD cognitive impairment progression, but markers for motor progression have been elusive^35–38^. We demonstrate that higher polygenic risk for PD is associated with short-term non-progression, perhaps operating through the known relationship between genetic risk and early-onset disease, and early-onset disease with slower progression^39,40^. Though in PDBP, where age of onset data is available, we observe no relationship between the PD polygenic risk score and age of clinical diagnosis, though age of clinical diagnosis may not capture the true time of disease onset, which in itself may be a continuum. Similarly, GWAS for age of PD onset appears to be overlapping with but distinct from PD diagnosis^41^. More notably, a right tail in the PD polygenic risk score distribution is apparent in both progressors and non-progressors. This right tail is driven by rs34637584(a) LRRK2 p.G2019S which has been previously associated with slower disease progression^33^.

In contrast, a higher polygenic score for educational attainment is associated with PD progression in meta-prediction, apparently through MDS-UPDRS III progression though the overall relationship with any individual subpart appears complex. Our rationale for the inclusion of the educational attainment PRS was that it may relate to the capacity to answer survey questions especially with advanced disease. In general, higher educational attainment is theorized to compensate for disease pathology, where a more robust cognitive or motor reserve allows for the tolerance to impairment despite more advanced disease pathology (42). Enhanced cognitive or motor reserve leading to lower apparent baseline impairment despite advanced disease fits the baseline condition for short-term disease progression. Though overall, the relationship between educational attainment polygenic scores and progression is not as straightforward as that for PD polygenic risk and potentially requires a closer dissection of the balance of cognitive vs motor reserves. One would expect motor and cognitive reserves would be least successful at compensating for findings revealed during the physician exam (MDS-UPDRS III), but it appears as an important predictor for all subparts. Interesting, while the educational attainment polygenic score is moderately correlated with educational status (*R*^2^ = 0.19), it is far from colinear and both the polygenic score and actual education level are included as important predictors. These predictors may potential act as measures of biological vs biological plus environmental cognitive reserve.

The limitations of our study include the enrichment of the PPMI cohort with monogenic early diagnosed cases relative to PDBP and the general population, the bias of individuals of European ancestry in both PPMI and PDBP relative to the general population, and the potential biased performance of polygenic scores in individuals of European ancestry relative to the general population. The major limitation is the heterogeneity of treatment status across the available cohort, the mixture of ON and OFF state MDS-UPDRS measurements used as a result, lack of dosage information for common PD treatments, and ultimately our inability to confidently control for the influence of medication effects which may influence MDS-UPDRS scores and progression status assignment. We attempted to adjust for medication status as best as the data would allow, though it remains possible that our results were influenced or confounded by differential medication effects across PD progressors and non-progressors. Additionally, treatment with dopamine agents may mask PD progression, and symptomatic medications for psychiatric, sleep and autonomic symptoms may affect the progression of MDS-UPDRS I scores, but granular information on these concomitant non-PD medications was not captured in the PPMI or PDBP datasets^2^.

Despite these limitations, overall, we demonstrate that short-term PD progression can be well predicted with a combination of survey-based, neuroimaging, physician examination, and genetic predictors in a meta-prediction framework. Meta-prediction can dissect the interplay between genetic risk, motor symptoms, non-motor symptoms, and longer-term expected rates of progression—potentially by capturing PD subtypes and their trajectories. Physician examination and polygenic risk scores provide the greatest predictive value in this framework. And finally, these predictions may enhance the efficiency of clinical trials by enriching them with individuals likely to demonstrate disease progression over the duration of a short clinical trial. For example, a well-powered (80%) randomized trial of an experimental therapeutic with 10% relative risk reduction in a population and a 50:50 split in progressors vs non-progressors, as observed in PDBP, would require just over 3000 study participants. If instead, screening of potential study participants enriched the trial to 75:25 progressors: non-progressors—then the required sample size would drop by nearly one-third. Thus, these predictions may be useful in accelerating the identification of PD disease modifying agents.

## Supplementary information

Supplemental Tables 1 - 5

Reporting Summary

Supplemental Tables

## Data Availability

Data was accessed and is available from AMP-PD (https://amp-pd.org/). Data was accessed under the standard AMP-PD data access agreement. Code used to generate our results is available here: https://github.com/drhosseinjavedani/Genetically-Informed-Prediction-of-Short-Term-Parkinson-Progression.
