# Supplementary material for: Genetically-informed prediction of short-term Parkinson’s disease progression
Source: NPJ Parkinsons Dis. 2022 Oct 28;8:143. doi: 10.1038/s41531-022-00412-w (PMC9613892; doi:10.1038/s41531-022-00412-w)
Supplement: Supplementary file 1 — Supplemental Tables 1 - 5 [file 41531_2022_412_MOESM1_ESM.pdf]

Supplemental Table 1

Treated at any point over 36 months

Treatment Status (%)	PPMI - UPDRS I			PDPB - UPDRS I		
	Progressors	Non-Progressors	P-value	Progressors	Non-Progressors	P-value
Levadopa	0.12 +/- 0.02	0.21 +/- 0.03	0.07	0.76 +/- 0.03	0.75 +/- 0.03	0.88
Dopamine Agonist	0.10 +/- 0.02	0.11 +/- 0.02	0.85	0.56 +/- 0.04	0.51 +/- 0.04	0.44
Other PD Medications	0.14 +/- 0.02	0.17 +/- 0.03	0.5	0.77 +/- 0.03	0.72 +/- 0.03	0.43
	PPMI - UPDRS II			PDPB - UPDRS II		
	Progressors	Non-Progressors	P-value	Progressors	Non-Progressors	P-value
Levadopa	0.13 +/- 0.02	0.19 +/- 0.03	0.24	0.76 +/- 0.03	0.75 +/- 0.03	0.92
Dopamine Agonist	0.11 +/- 0.02	0.11 +/- 0.02	0.95	0.57 +/- 0.04	0.49 +/- 0.04	0.22
Other PD Medications	0.13 +/- 0.02	0.18 +/- 0.03	0.36	0.76 +/- 0.03	0.72+/- 0.03	0.59
	PPMI - UPDRS III			PDPB - UPDRS III		
	Progressors	Non-Progressors	P-value	Progressors	Non-Progressors	P-value
Levadopa	0.13 +/- 0.02	0.20 +/- 0.03	0.19	0.75 +/- 0.03	0.76 +/- 0.03	0.95
Dopamine Agonist	0.09 +/- 0.02	0.14 +/- 0.02	0.29	0.54 +/- 0.04	0.52 +/- 0.04	0.84
Other PD Medications	0.13 +/- 0.02	0.18 +/- 0.03	0.38	0.75 +/- 0.03	0.74 +/- 0.03	0.86

Treatment status at month 12

Treatment Status (%)	PPMI - UPDRS I			PDPB - UPDRS I		
	Progressors	Non-Progressors	P-value	Progressors	Non-Progressors	P-value
Levadopa	0.14 +/-0.01	0.22 +/-0.02	0.07	0.75 +/-0.03	0.74 +/-0.03	0.86
Dopamine Agonist	0.10 +/-0.01	0.11 +/-0.01	0.89	0.55 +/-0.03	0.52 +/-0.03	0.59
Other PD Medications	0.14 +/-0.01	0.18 +/-0.02	0.39	0.77+/-0.03	0.71+/-0.03	0.38
	PPMI - UPDRS II			PDPB - UPDRS II		
	Progressors	Non-Progressors	P-value	Progressors	Non-Progressors	P-value
Levadopa	0.15 +/-0.01	0.22 +/-0.02	0.14	0.75 +/-0.03	0.74 +/-0.03	0.89
Dopamine Agonist	0.10 +/-0.01	0.11 +/-0.02	0.76	0.58 +/-0.03	0.50 +/-0.03	0.14
Other PD Medications	0.14 +/-0.01	0.19 +/-0.02	0.29	0.76 +/-0.03	0.75 +/-0.01	0.51
	PPMI - UPDRS III			PDPB - UPDRS III		
	Progressors	Non-Progressors	P-value	Progressors	Non-Progressors	P-value
Levadopa	0.16 +/-0.01	0.20 +/-0.02	0.37	0.75 +/-0.03	0.74 +/-0.03	0.79
Dopamine Agonist	0.08 +/-0.01	0.14 +/-0.01	0.22	0.54 +/-0.03	0.53 +/-0.03	0.82
Other PD Medications	0.15 +/-0.01	0.18 +/-0.02	0.55	0.76 +/-0.03	0.73 +/-0.03	0.67

Treatment status at month 24

Treatment Status (%)	PPMI - UPDRS I			PDPB - UPDRS I		
	Progressors	Non-Progressors	P-value	Progressors	Non-Progressors	P-value
Levadopa	0.13 +/-0.01	0.26 +/-0.02	0.007	0.74 +/-0.03	0.75+/-0.03	0.96
Dopamine Agonist	0.09 +/-0.01	0.12 +/-0.02	0.52	0.54 +/-0.03	0.53 +/-0.03	0.9
Other PD Medications	0.13+/-0.01	0.21+/-0.02	0.076	0.76 +/-0.03	0.72 +/-0.03	0.5
	PPMI - UPDRS II			PDPB - UPDRS II		
	Progressors	Non-Progressors	P-value	Progressors	Non-Progressors	P-value
Levadopa	0.17 +/-0.01	0.19 +/-0.02	0.6	0.77 +/-0.02	0.71 +/-0.03	0.26
Dopamine Agonist	0.10 +/-0.01	0.11 +/-0.02	0.79	0.55 +/-0.03	0.52 +/-0.03	0.7
Other PD Medications	0.15 +/-0.01	0.18 +/-0.02	0.58	0.76 +/-0.02	0.71 +/-0.03	0.34
	PPMI - UPDRS III			PDPB - UPDRS III		
	Progressors	Non-Progressors	P-value	Progressors	Non-Progressors	P-value
Levadopa	0.19 +/-0.01	0.16 +/-0.02	0.61	0.74 +/-0.03	0.75 +/-0.03	0.79
Dopamine Agonist	0.10 +/-0.01	0.11 +/-0.02	0.82	0.54 +/-0.03	0.53 +/-0.03	0.75
Other PD Medications	0.16 +/-0.01	0.15 +/-0.02	0.81	0.76 +/-0.02	0.73 +/-0.03	0.52

Treatment status at month 36

Treatment Status (%)	PPMI - UPDRS I			PDPB - UPDRS I		
	Progressors	Non-Progressors	P-value	Progressors	Non-Progressors	P-value
Levadopa	0.14 +/-0.01	0.26 +/-0.03	0.01	0.74 +/-0.02	0.75+/-0.03	0.93
Dopamine Agonist	0.09 +/-0.01	0.13 +/-0.02	0.45	0.51 +/-0.03	0.59 +/-0.04	0.17
Other PD Medications	0.12+/-0.01	0.24+/-0.03	0.015	0.76 +/-0.02	0.70 +/-0.03	0.29
	PPMI - UPDRS II			PDPB - UPDRS II		
	Progressors	Non-Progressors	P-value	Progressors	Non-Progressors	P-value
Levadopa	0.18 +/-0.01	0.18 +/-0.02	0.97	0.80 +/-0.02	0.66 +/-0.03	0.2
Dopamine Agonist	0.12 +/-0.01	0.07 +/-0.01	0.27	0.52 +/-0.03	0.57 +/-0.04	0.37
Other PD Medications	0.16 +/-0.01	0.16 +/-0.02	0.98	0.76 +/-0.02	0.70 +/-0.03	0.27
	PPMI - UPDRS III			PDPB - UPDRS III		
	Progressors	Non-Progressors	P-value	Progressors	Non-Progressors	P-value
Levadopa	0.19 +/-0.02	0.16 +/-0.02	0.58	0.75 +/-0.02	0.74 +/-0.03	0.91
Dopamine Agonist	0.10 +/-0.01	0.11 +/-0.02	0.96	0.54 +/-0.03	0.53 +/-0.04	0.79
Other PD Medications	0.17 +/-0.01	0.14 +/-0.02	0.57	0.75 +/-0.02	0.71 +/-0.03	0.48

Supplemental Table 2

Feature	Average in Progressors +/- Standard Error	Average in None-Progressors +/- Standard Error	P-value
pd_diagnosis_months_after_baseline	-7.3882 +/- 0.45	-7.5894+/-0.64	0.017
age_at_baseline	62.1423 +/- 0.61	61.4516+/-0.88	0.033
enrollment_months_after_baseline	-0.5732 +/- 0.02	-0.5887+/-0.03	0.038
moca01_alternating_trail_making	0.8984 +/- 0.02	0.904+/-0.03	0.018
moca02_visuoconstr_skills_cube	0.8089 +/- 0.03	0.784+/-0.04	0.036
moca03_visuoconstr_skills_clock_cont	0.9919 +/- 0.01	0.976+/-0.01	0.025
moca04_visuoconstr_skills_clock_num	0.9512 +/- 0.01	0.952+/-0.02	0.016
moca05_visuoconstr_skills_clock_hands	0.8374 +/- 0.02	0.848+/-0.03	0.016
moca_visuospatial_executive_subscore	4.4878 +/- 0.05	4.464+/-0.08	0.018
moca06_naming_lion	0.9919 +/- 0.01	1.0+/-0.0	0.013
moca07_naming_rhino	0.9553 +/- 0.01	0.952+/-0.02	0.018
moca08_naming_camel	1.0 +/- 0.0	0.976+/-0.01	0.03
moca_naming_subscore	2.9472 +/- 0.01	2.928+/-0.03	0.024
moca09_attention_forward_digit_span	0.9756 +/- 0.01	0.976+/-0.01	0.017
moca10_attention_backward_digit_span	0.9309 +/- 0.02	0.968+/-0.02	0.007
moca_attention_digits_subscore	1.9065 +/- 0.02	1.944+/-0.02	0.007
moca11_attention_vigilance	0.9878 +/- 0.01	0.984+/-0.01	0.018
moca12_attention_serial_7s	2.874 +/- 0.03	2.848+/-0.05	0.012
moca13_sentence_repetition	1.7602 +/- 0.03	1.864+/-0.03	0.002
moca14_verbal_fluency_number_of_words	13.3008 +/- 0.3	12.808+/-0.44	0.041
moca15_verbal_fluency	0.7724 +/- 0.03	0.768+/-0.04	0.022
moca_language_subscore	2.5325 +/- 0.04	2.632+/-0.05	0.007
moca16_abstraction	1.8821 +/- 0.02	1.856+/-0.04	0.018
moca_abstraction_subscore	1.8821 +/- 0.02	1.856+/-0.04	0.018
moca17_delayed_recall_face	0.5755 +/- 0.03	0.5565+/-0.04	0.027
moca18_delayed_recall_velvet	0.7592 +/- 0.03	0.8306+/-0.03	0.001
moca19_delayed_recall_church	0.7114 +/- 0.03	0.7581+/-0.04	0.023
moca20_delayed_recall_daisy	0.6016 +/- 0.03	0.6129+/-0.04	0.049
moca21_delayed_recall_red	0.7358 +/- 0.03	0.736+/-0.04	0.028
moca_delayed_recall_subscore	3.378 +/- 0.09	3.472+/-0.11	0.012
moca22_orientation_date_score	0.9837 +/- 0.01	0.952+/-0.02	0.036
moca23_orientation_month_score	1.0 +/- 0.0	1.0+/-0.0	0.016
moca25_orientation_day_score	0.9919 +/- 0.01	0.992+/-0.01	0.017
moca26_orientation_place_score	0.9959 +/- 0.0	1.0+/-0.0	0.014
moca27_orientation_city_score	1.0 +/- 0.0	1.0+/-0.0	0.016
moca_orientation_subscore	5.9715 +/- 0.01	5.944+/-0.02	0.03
moca_total_score	27.1423 +/- 0.15	27.248+/-0.2	0.013
mod_schwab_england_pct_adl_score	93.9941 +/- 0.42	93.6508+/-0.52	0.478
sbr_caudate_r	2.1111 +/- 0.04	2.0339+/-0.06	0.06
sbr_caudate_l	2.107 +/- 0.04	2.0387+/-0.06	0.048
sbr_putamen_r	0.9704 +/- 0.03	0.9971+/-0.05	0.02
sbr_putamen_l	0.9347 +/- 0.03	0.9613+/-0.05	0.018
parkinsons_disease_PRS	0.0046 +/- 0.0	0.0054+/-0.0	0.229
educational_attainment_PRS	0.0005 +/- 0.0	0.0004+/-0.0	0.594
upd2301_speech_problems	1.4366 +/- 0.03	1.5684+/-0.04	0.027
upd2302_facial_expression	2.0649 +/- 0.04	2.1632+/-0.06	0.178
upd2303a_rigidity_neck	1.5693 +/- 0.04	1.7737+/-0.06	0.009
upd2303b_rigidity_rt_upper_extremity	1.9528 +/- 0.05	2.0368+/-0.06	0.311
upd2303c_rigidity_left_upper_extremity	1.7611 +/- 0.05	1.9789+/-0.06	0.006
upd2303d_rigidity_rt_lower_extremity	1.5988 +/- 0.04	1.5895+/-0.06	0.833
upd2303e_rigidity_left_lower_extremity	1.4661 +/- 0.04	1.6158+/-0.06	0.075
upd2304a_right_finger_tapping	1.9912 +/- 0.04	2.2789+/-0.07	0.001
upd2304b_left_finger_tapping	1.9115 +/- 0.05	2.2737+/-0.07	0
upd2305a_right_hand_movements	1.7493 +/- 0.04	1.9579+/-0.06	0.011
upd2305b_left_hand_movements	1.7817 +/- 0.05	2.0105+/-0.07	0.005
upd2306a_pron_sup_movement_right_hand	1.7198 +/- 0.04	1.9474+/-0.06	0.006
upd2306b_pron_sup_movement_left_hand	1.7375 +/- 0.05	2.0053+/-0.07	0.001
upd2307a_right_toe_tapping	1.7286 +/- 0.04	2.0579+/-0.07	0
upd2307b_left_toe_tapping	1.8112 +/- 0.05	2.2421+/-0.07	0
upd2308a_right_leg_agility	1.4395 +/- 0.03	1.7316+/-0.06	0
upd2308b_left_leg_agility	1.4956 +/- 0.04	1.7632+/-0.06	0
upd2309_arising_from_chair	1.1829 +/- 0.02	1.2421+/-0.04	0.302
upd2310_gait	1.5516 +/- 0.03	1.6947+/-0.05	0.026
upd2311_freezing_of_gait	1.0177 +/- 0.01	1.0316+/-0.02	0.903
upd2312_postural_stability	1.1239 +/- 0.02	1.2526+/-0.05	0.205
upd2313_posture	1.5988 +/- 0.04	1.7+/-0.05	0.139
upd2314_body_bradykinesia	2.1799 +/- 0.04	2.3211+/-0.06	0.083
upd2315a_postural_tremor_of_right_hand	1.4189 +/- 0.03	1.4474+/-0.04	0.494
upd2315b_postural_tremor_of_left_hand	1.354 +/- 0.03	1.3737+/-0.04	0.481
upd2316a_kinetic_tremor_of_right_hand	1.3776 +/- 0.03	1.3789+/-0.04	0.83
upd2316b_kinetic_tremor_of_left_hand	1.3923 +/- 0.03	1.3789+/-0.04	0.833
upd2317a_rest_tremor_amplitude_right_upper_extremity	1.6195 +/- 0.05	1.6053+/-0.06	0.911
upd2317b_rest_tremor_amplitude_left_upper_extremity	1.4277 +/- 0.04	1.4579+/-0.05	0.646
upd2317c_rest_tremor_amplitude_right_lower_extremity	1.1386 +/- 0.02	1.2053+/-0.04	0.627
upd2317d_rest_tremor_amplitude_left_lower_extremity	1.1062 +/- 0.02	1.1895+/-0.04	0.254
upd2317e_rest_tremor_amplitude_lip_or_jaw	1.0531 +/- 0.01	1.0895+/-0.02	0.609
upd2318_consistency_of_rest_tremor	2.1593 +/- 0.06	2.2579+/-0.09	0.576
upd2hy_hoehn_and_yahr_stage	1.5546 +/- 0.03	1.7632+/-0.04	0
upd2201_speech	1.4543 +/- 0.04	1.5737+/-0.05	0.048
upd2202_saliva_and_drooling	1.587 +/- 0.05	1.7421+/-0.08	0.066
upd2203_chewing_and_swallowing	1.177 +/- 0.03	1.1947+/-0.04	0.813
upd2204_eating_tasks	1.3392 +/- 0.03	1.3684+/-0.04	0.651
upd2205_dressing	1.4602 +/- 0.03	1.5+/-0.05	0.431
upd2206_hygiene	1.2448 +/- 0.02	1.2368+/-0.03	0.973
upd2207_handwriting	1.8732 +/- 0.05	1.9737+/-0.07	0.424
upd2208_doing_hobbies_and_other_activities	1.4513 +/- 0.04	1.6053+/-0.06	0.009
upd2209_turning_in_bed	1.3451 +/- 0.03	1.3421+/-0.04	0.892
upd2210_tremor	2.0383 +/- 0.04	2.0947+/-0.05	0.439
upd2211_get_out_of_bed_car_or_deep_chair	1.4779 +/- 0.03	1.5421+/-0.05	0.39
upd2212_walking_and_balance	1.4277 +/- 0.03	1.5526+/-0.05	0.046
upd2213_freezing	1.0944 +/- 0.02	1.1263+/-0.03	0.763
upd2101_cognitive_impairment	1.3068 +/- 0.03	1.3632+/-0.04	0.239
upd2102_hallucinations_and_psychosis	1.056 +/- 0.01	1.0474+/-0.02	0.882
upd2103_depressed_mood	1.3186 +/- 0.03	1.4421+/-0.06	0.314
upd2104_anxious_mood	1.4159 +/- 0.03	1.6053+/-0.06	0.027
upd2105_apathy	1.2183 +/- 0.03	1.2895+/-0.05	0.438
upd2106_dopamine_dysregulation_syndrome_features	1.0324 +/- 0.01	1.0579+/-0.02	0.843
upd2107_pat_quest_sleep_problems	1.9056 +/- 0.06	2.1421+/-0.08	0.026
upd2108_pat_quest_daytime_sleepiness	1.8171 +/- 0.05	1.8263+/-0.06	0.857
upd2109_pat_quest_pain_and_other_sensations	1.767 +/- 0.05	1.7895+/-0.07	0.73
upd2110_pat_quest_urinary_problems	1.6431 +/- 0.05	1.6737+/-0.06	0.759
upd2111_pat_quest_constipation_problems	1.4956 +/- 0.04	1.5+/-0.06	0.985
upd2112_pat_quest_lightheadedness_on_standing	1.3628 +/- 0.03	1.4+/-0.05	0.576
upd2113_pat_quest_fatigue	1.7109 +/- 0.05	1.8+/-0.06	0.226
mds_updrs_part_i_sub_score	1.3481 +/- 0.1	1.8466+/-0.17	0.009
mds_updrs_part_i_pat_quest_sub_score	4.7021 +/- 0.2	5.1958+/-0.25	0.021
mds_updrs_part_i_summary_score	4.16 +/- 0.26	4.6384+/-0.36	0.204
mds_updrs_part_ii_summary_score	3.5721 +/- 0.25	4.2608+/-0.34	0.043
mds_updrs_part_iii_summary_score	15.9481 +/- 0.53	21.0221+/-0.73	0
mds_updrs_all	23.6803 +/- 0.8	29.9175+/-1.07	0

Supplemental Table 3

Feature	Average in Progressors +/- Standard Error	Average in None Progressors +/- Standard Error	P-values
age_at_baseline	64.9118 +/- 0.72	65.3833+/-0.64	0.745
age_at_diagnosis	59.4071 +/- 1.15	60.0283+/-1.01	0.034
enrollment_months_after_baseline	-0.5579 +/- 0.03	-0.5398+/-0.03	0.958
moca_visuospatial_executive_subscore	4.1941 +/- 0.09	4.2849+/-0.08	0.665
moca_naming_subscore	2.8941 +/- 0.03	2.8939+/-0.02	0.999
moca_attention_digits_subscore	1.8588 +/- 0.03	1.8492+/-0.03	0.873
moca11_attention_vigilance	0.9471 +/- 0.02	0.9553+/-0.02	0.824
moca12_attention_serial_7s	2.8118 +/- 0.04	2.7697+/-0.04	0.613
moca13_sentence_repetition	1.6154 +/- 0.04	1.6648+/-0.04	0.471
moca15_verbal_fluency	0.6647 +/- 0.04	0.7458+/-0.03	0.119
moca_language_subscore	2.2706 +/- 0.06	2.4022+/-0.06	0.134
moca16_abstraction	1.8817 +/- 0.03	1.8202+/-0.03	0.419
moca_abstraction_subscore	1.8817 +/- 0.03	1.8202+/-0.03	0.419
moca_delayed_recall_subscore	3.2485 +/- 0.11	3.1966+/-0.12	0.976
moca_orientation_subscore	5.8706 +/- 0.05	5.8827+/-0.04	0.987
moca_score	26.0529 +/- 0.24	26.1341+/-0.25	0.628
mod_schwab_england_pct_adl_score	88.4706 +/- 0.93	87.4157+/-1.02	0.568
parkinsons_disease_PRS	0.0026 +/- 0.0	0.0025+/-0.0	0.534
educational_attainment_PRS	0.0005 +/- 0.0	0.0004+/-0.0	0.091
upd2301_speech_problems	1.5706 +/- 0.06	1.6556+/-0.06	0.301
upd2302_facial_expression	1.6941 +/- 0.06	1.8222+/-0.06	0.088
upd2303a_rigidity_neck	1.7235 +/- 0.07	2.1111+/-0.08	0.001
upd2303b_rigidity_rt_upper_extremity	2.0882 +/- 0.06	2.3444+/-0.06	0.005
upd2303c_rigidity_left_upper_extremity	2.0647 +/- 0.07	2.2333+/-0.07	0.063
upd2303d_rigidity_rt_lower_extremity	1.6118 +/- 0.07	1.85+/-0.07	0.011
upd2303e_rigidity_left_lower_extremity	1.5647 +/- 0.07	1.8+/-0.07	0.017
upd2304a_right_finger_tapping	2.0118 +/- 0.07	2.2278+/-0.06	0.014
upd2304b_left_finger_tapping	2.1353 +/- 0.07	2.4+/-0.07	0.007
upd2305a_right_hand_movements	1.8294 +/- 0.06	1.9667+/-0.07	0.264
upd2305b_left_hand_movements	1.9471 +/- 0.07	2.1944+/-0.07	0.023
upd2306a_pron_sup_movement_right_hand	1.8059 +/- 0.07	1.9778+/-0.07	0.068
upd2306b_pron_sup_movement_left_hand	2.0176 +/- 0.08	2.2278+/-0.07	0.019
upd2307a_right_toe_tapping	1.7824 +/- 0.07	2.0333+/-0.07	0.01
upd2307b_left_toe_tapping	1.9647 +/- 0.07	2.2778+/-0.08	0.008
upd2308a_right_leg_agility	1.3941 +/- 0.05	1.5889+/-0.06	0.081
upd2308b_left_leg_agility	1.5118 +/- 0.06	1.6667+/-0.07	0.252
upd2309_arising_from_chair	1.2118 +/- 0.04	1.2667+/-0.05	0.752
upd2310_gait	1.6941 +/- 0.05	1.8389+/-0.05	0.09
upd2311_freezing_of_gait	1.0824 +/- 0.03	1.1833+/-0.04	0.343
upd2312_postural_stability	1.4471 +/- 0.07	1.4944+/-0.07	0.743
upd2313_posture	1.6588 +/- 0.07	1.8056+/-0.07	0.187
upd2314_body_bradykinesia	1.8059 +/- 0.05	1.9444+/-0.06	0.282
upd2315a_postural_tremor_of_right_hand	1.4 +/- 0.05	1.5333+/-0.05	0.114
upd2315b_postural_tremor_of_left_hand	1.4412 +/- 0.05	1.5444+/-0.05	0.181
upd2316a_kinetic_tremor_of_right_hand	1.6059 +/- 0.05	1.6833+/-0.05	0.4
upd2316b_kinetic_tremor_of_left_hand	1.7059 +/- 0.05	1.7944+/-0.05	0.328
upd2317a_rest_tremor_amplitude_right_upper_extremity	1.3353 +/- 0.05	1.4944+/-0.06	0.2
upd2317b_rest_tremor_amplitude_left_upper_extremity	1.3529 +/- 0.06	1.45+/-0.06	0.213
upd2317c_rest_tremor_amplitude_right_lower_extremity	1.0647 +/- 0.02	1.1167+/-0.03	0.449
upd2317d_rest_tremor_amplitude_left_lower_extremity	1.0765 +/- 0.03	1.1278+/-0.03	0.561
upd2317e_rest_tremor_amplitude_lip_or_jaw	1.0294 +/- 0.01	1.0722+/-0.02	0.667
upd2318_consistency_of_rest_tremor	1.8824 +/- 0.1	2.2722+/-0.11	0.005
upd2hy_hoehn_and_yahr_stage	2.0353 +/- 0.04	2.1167+/-0.05	0.415
upd2201_speech	1.7059 +/- 0.07	1.7556+/-0.07	0.806
upd2202_saliva_and_drooling	1.9882 +/- 0.09	2.0111+/-0.09	0.963
upd2203_chewing_and_swallowing	1.2235 +/- 0.04	1.3111+/-0.04	0.096
upd2204_eating_tasks	1.3824 +/- 0.05	1.5722+/-0.06	0.049
upd2205_dressing	1.6353 +/- 0.06	1.7722+/-0.06	0.169
upd2206_hygiene	1.2706 +/- 0.04	1.4+/-0.05	0.062
upd2207_handwriting	2.0824 +/- 0.09	2.2222+/-0.09	0.249
upd2208_doing_hobbies_and_other_activities	1.7235 +/- 0.08	1.7889+/-0.08	0.599
upd2209_turning_in_bed	1.4824 +/- 0.05	1.6667+/-0.07	0.143
upd2210_tremor	2.0118 +/- 0.06	2.2278+/-0.06	0.03
upd2211_get_out_of_bed_car_or_deep_chair	1.7412 +/- 0.06	1.8222+/-0.06	0.379
upd2212_walking_and_balance	1.8588 +/- 0.08	1.7056+/-0.07	0.163
upd2213_freezing	1.3882 +/- 0.07	1.35+/-0.06	0.936
upd2101_cognitive_impairment	1.4824 +/- 0.06	1.5278+/-0.07	0.859
upd2102_hallucinations_and_psychosis	1.1529 +/- 0.04	1.1222+/-0.03	0.951
upd2103_depressed_mood	1.2882 +/- 0.05	1.25+/-0.05	0.474
upd2104_anxious_mood	1.4412 +/- 0.06	1.4944+/-0.06	0.379
upd2105_apathy	1.2235 +/- 0.04	1.2278+/-0.04	0.864
upd2106_dopamine_dysregulation_syndrome_features	1.1294 +/- 0.03	1.1333+/-0.03	0.929
mds_updrs_part_i_sub_score	1.7176 +/- 0.18	1.7556+/-0.17	0.854
upd2107_pat_quest_sleep_problems	2.2706 +/- 0.09	2.2944+/-0.09	0.826
upd2108_pat_quest_daytime_sleepiness	2.1 +/- 0.07	2.2389+/-0.07	0.241
upd2109_pat_quest_pain_and_other_sensations	1.9824 +/- 0.08	2.0222+/-0.08	0.641
upd2110_pat_quest_urinary_problems	1.8118 +/- 0.08	1.9667+/-0.08	0.229
upd2111_pat_quest_constipation_problems	1.6706 +/- 0.07	1.8222+/-0.07	0.038
upd2112_pat_quest_lightheadedness_on_standing	1.4471 +/- 0.06	1.55+/-0.07	0.452
upd2113_pat_quest_fatigue	1.8882 +/- 0.06	2.0278+/-0.08	0.459
mds_updrs_part_i_pat_quest_sub_score	6.1706 +/- 0.32	6.9222+/-0.32	0.11
mds_updrs_part_i_summary_score	4.1824 +/- 0.39	4.6854+/-0.43	0.566
mds_updrs_part_ii_summary_score	4.7581 +/- 0.5	5.6231+/-0.56	0.355
mds_updrs_part_iii_summary_score	19.1496 +/- 0.94	24.4997+/-1.05	0
mds_updrs_all	28.0902 +/- 1.51	34.8082+/-1.71	0.002

Supplemental Table 4

Full Meta-prediction

	PPMI			PDPB	
	Non-Progressors	Progressors		Non-Progressors	Progressors
Non-Progressors	25	22		130	52
Progressors	18	67		37	131

No Genetics

	PPMI			PDPB	
	Non-Progressors	Progressors		Non-Progressors	Progressors
Non-Progressors	18	29		120	62
Progressors	28	57		50	118

No Physician Exam

	PPMI			PDPB	
	Non-Progressors	Progressors		Non-Progressors	Progressors
Non-Progressors	21	26		126	56
Progressors	25	60		46	122

No Surveys

	PPMI			PDPB	
	Non-Progressors	Progressors		Non-Progressors	Progressors
Non-Progressors	23	24		125	57
Progressors	23	62		39	129

Supplemental Table 5

			PPMI		
	Sensitivity	Specificity	Positive Predictive Value	Negative Predictive	F1-score
**Full Meta-prediction**	0.79	0.53	0.75	0.58	0.77
**No Genetics**	0.67	0.38	0.66	0.39	0.67
**No Physician Exam**	0.71	0.45	0.7	0.46	0.7
**No Surveys**	0.73	0.49	0.72	0.5	0.73

			PDBP		
	Sensitivity	Specificity	Positive Predictive Value	Negative Predictive	F1-score
**Full Meta-prediction**	0.78	0.71	0.72	0.78	0.75
**No Genetics**	0.7	0.66	0.66	0.71	0.68
**No Physician Exam**	0.73	0.69	0.69	0.73	0.71
**No Surveys**	0.77	0.69	0.69	0.76	0.73
