# Supplementary material for: Genetically-informed prediction of short-term Parkinson’s disease progression
Source: NPJ Parkinsons Dis. 2022 Oct 28;8:143. doi: 10.1038/s41531-022-00412-w (PMC9613892; doi:10.1038/s41531-022-00412-w)
Supplement: Supplementary file 3 — Supplemental Tables [file 41531_2022_412_MOESM3_ESM.docx]

**Supplemental Tables**

**Supplemental Table 1.** Rate of medication usage in PPMI and PDBP by MDS-UPDRS subpart progression status.

**Supplemental Table 2.** Detailed Baseline Characteristics of 12-month MDS-UPDRS Total Progressors vs Non-Progressors for PPMI

**Supplemental Table 3.** Detailed Baseline Characteristics of 12-month MDS-UPDRS Total Progressors vs Non-Progressors for PDBP.

**Supplemental Table 4.** Confusion matrices for full meta-prediction and with feature class removal. Note that reported PPMI numbers are not the full cohort but from the final train-test split used for feature testing.

**Supplemental Table 5.** Additional accuracy metrics.
